# Non-Hodgkin’s Lymphoma and Colorectal Carcinoma: Metachronous Occurrence in a Patient With Underlying DNA Mismatch Repair Syndrome

**DOI:** 10.7759/cureus.12092

**Published:** 2020-12-15

**Authors:** Zunaira Shaukat, Rabia Wali

**Affiliations:** 1 Pediatric Oncology, Shaukat Khanum Memorial Cancer Hospital and Research Centre, Lahore, PAK

**Keywords:** colorectal neoplasms, constitutional mismatch repair deficiency, dna mismatch repair, lymphoma, non-hodgkin's, lynch syndrome

## Abstract

Constitutional mismatch repair deficiency (CMMRD) is an autosomal recessive disorder caused by biallelic mutations in DNA mismatch repair genes 1. These patients have clinical stigmata of neurofibromatosis 1 (NF-1) with childhood onset of hematological malignancies, high grade gliomas, and colorectal-cancers 2. We present a case of non-Hodgkin's lymphoma (NHL) who later on developed adenocarcinoma colon at an age of 11 years with significant family history of glioblastoma in elder brother and colonic cancer in mother. This is the first case of CMMRD in Pakistan who developed colonic neoplasm at the age of 11 years. Nearly 150 patients of CMMRD have been reported worldwide.

## Introduction

Constitutional mismatch repair deficiency (CMMRD) is a rare cancer predisposition syndrome that results from biallelic mutations in mismatch repair genes [1-2]. This disorder is similar to Lynch syndrome (LS) that manifests usually in young adults. LS is an autosomal dominant disorder caused by defects in one of DNA MMR genes. Siblings of two parents with LS can develop CMMRD (biallelic MMR mutations). The spectrum of cancers observed for CMMRD are more severe than those found in LS. CMMRD results in progressive accumulation of truncated proteins in the cells leading to a variety of LS related malignancies that occur in meta-chronous or synchronous fashion in the same individual [3]. 

Awareness of CMMRD is needed especially in pediatric oncologists as well as general pediatricians because these children often present with clinical stigmata of neurofibromatosis 1 (NF 1) [1-2]. Patients with NF 1 are also at high risk of developing cancers but they do so usually in teenage or young adulthood. In contrast, patients with CMMRD develop cancers from infancy. So surveillance programs for such patients should be initiated early in life [1-3]. 

## Case presentation

**Figure 1:**
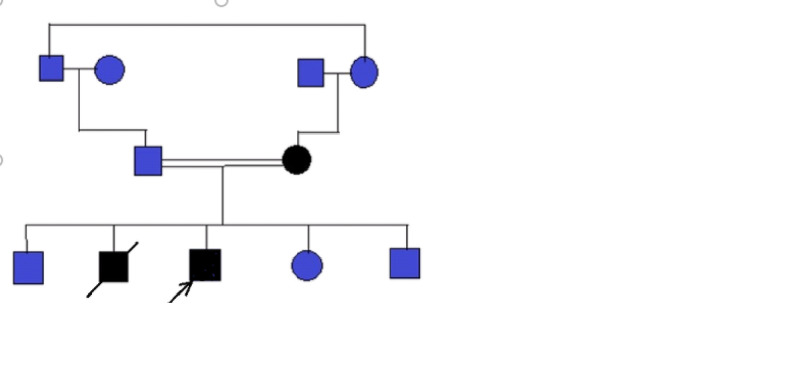
Pedigree. Index case was the product of consanguineous marriage. Mother developed colorectal carcinoma at the age of 40 years. Elder brother died of GBM at the age of seven years. Index case diagnosed with NHL at six years of age and developed adenocarcinoma colon at the age of 11 years.  Arrow indicates index case. Black shaded boxes are affected individuals in family. NHL, non-Hodgkin's lymphoma; GBM, glioblastoma multiforme

A six-year-old boy presented in 2014 with huge neck and anterior chest wall mass. On examination, he had multiple Café-au-lait spots on trunk. He was diagnosed as T cell lymphoblastic lymphoma (LBL) on cervical lymph node biopsy. Family history was significant as his elder brother died of glioblastoma multiforme at the age of seven years and his mother developed colorectal carcinoma at the age of 40 years (Figure 1).

**Figure 2:**
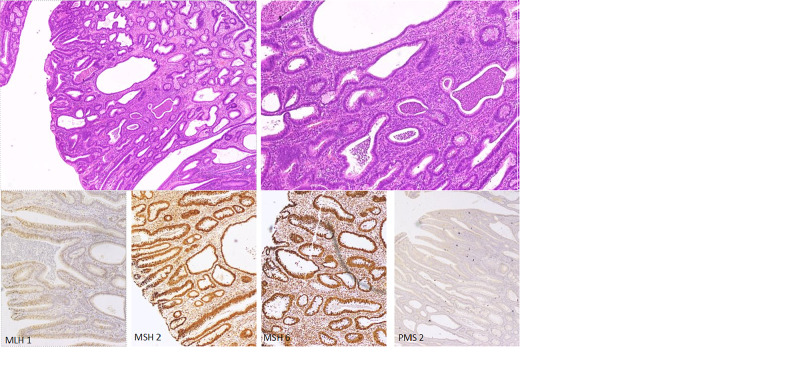
Histopathology of colonic polyp. H&E staining of colonic polyp showing moderately differentiated adenocarcinoma arising in the background of tubulo-villous adenoma (transverse colon), 11 mm. Immunostaining retained for MSH2 and MSH 6 in colonic polyp. Immunostaining lost in all cells for MLH1 and PMS2.

 He received treatment as per UKALL 2011 Regimen C [4]. The patient initially developed signs of superior vena cava obstruction syndrome and tumor lysis syndrome that were managed in ICU. End induction CT scan was in morphological remission. He finished his treatment in September 2017. He was disease free and remained on follow up. In late 2018, he presented with hematochezia and rectal prolapse that were initially managed conservatively. Later on, for persistent PR bleeding, diagnostic colonoscopy performed in 2019 that showed multiple variable sized polyps, measuring around 2-4 cm, scattered throughout the transverse colon, descending and sigmoid colon and removed as a two-stage procedure. Histopathology revealed tubulo-villous adenoma in one of the excised polyps. At that point underlying cancer predisposition syndrome was suspected. Given the strong family history of malignancy and predisposition to have further cancer it was recommended to do total colectomy after multi-disciplinary discussion. The patient underwent total colectomy with ileoanal anastomosis. Histopathology of total colectomy and excised lymph nodes showed moderately differentiated adenocarcinoma arising in the background of tubulo-villous adenoma in transverse colon (Figure 2). There were multiple adenomatous polyps in the resected colon while one out of 44 lymph nodes were positive for metastatic carcinoma without extra nodal extension (pT1, pN1a, M0). Immunohistochemistry (IHC) testing for mismatch repair (MMR) proteins was performed with intact expression of MSH2, MSH6 and loss of PMS2 and MLH-1 expression (Figure 2). 

**Figure 3:**
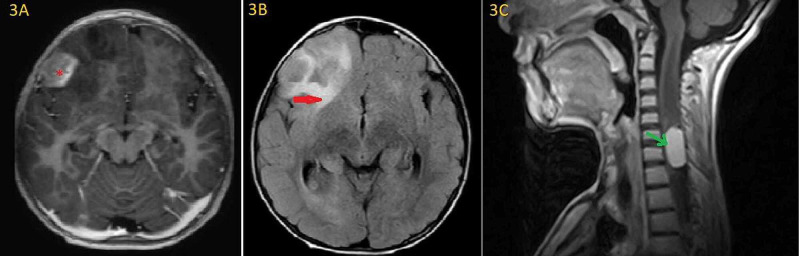
MRI brain and cervical spine. A: Heterogeneously enhancing cortical and subcortical lesions in the right frontal lobe (red asterisk) with associated surrounding vasogenic edema (red arrow) causing complete effacement of the anterior horn of right lateral ventricle (B). C: MRI cervical spine shows homogenously enhancing extra-axial dural-based abnormality in the spinal canal at the level of C7-T2 vertebral bodies suggestive of meningioma (green arrow).

This was the second malignancy arising in the same patient documented about two years after finishing treatment for first cancer. Father was offered adjuvant chemotherapy as per FOLFOX4 regimen but he refused to continue with chemotherapy treatment in curative intent. The patient remained on close follow up for about four months and then developed left-sided hemiparesis in November 2019. Brain and spine MRI done showed multiple T2 isointense, heterogeneously enhancing lesion in the right frontal lobe (Figure 3A) causing mass effect (Figure 3B). MRI cervical spine revealed 26 mm x 12 mm homogenously enhancing extra-axial dural-based abnormality in the spinal canal at the level of C7-T2 vertebral bodies suggestive of meningioma (Figure 3C). 

Biopsy of frontal lobe lesion was planned but parents refused any sort of further interventions and curative treatment. Unfortunately, the patient died one month later following a convulsion at home. Parents were offered genetic counseling and sibling screening but they did not seek it. His tissue samples were sent for analysis in CMMRD consortium and there was a high tumor mutation burden of 147 mut/mb with a POLE mutation (p.A456D) in his tissue which is a rare mutation. Family screening for this specific mutation and future surveillance is planned.

## Discussion

Constitutional mismatch repair deficiency is an autosomal recessive condition, which is similar to LS with earlier presentation of LS associated cancers during childhood. LS is an autosomal dominant disorder with incomplete penetrance, caused by mutation in any one allele among DNA mismatch repair genes [3]. It usually presents in young adult age while CMMRD is caused by biallelic mutation in one of the LS related genes and can present as early as infancy with hematological malignancies. The most common malignancies that occur are non-Hodgkin's lymphoma (median age: 6.6 years) [5], glioblastoma multiforme (median age: 10.3 years) [6] and colonic neoplasms and many more diverse cancer types. The genes involved in the development of CMMRD syndrome and LS are MLH1, MSH2, MSH6, and PMS2. These tumors are hypermutated and contain neo-antigens that can be targets for future therapies [7]. Rapid diagnosis of this condition needs high index of suspicion and awareness among physicians. This is important to tailor treatment as per underlying MMR defect, future surveillance, and screening of affected family members. European “care for CMMRD” consortium has developed a diagnostic tool for these patients.

The CMMRD should be suspected if a patient has combination of cancers belonging to the spectrum (T-NHL, malignant gliomas, colonic cancers) and skin manifestations of Cafe au lait macules or hypo/hyper pigmented spots. According to current scoring system, patients with ≥3 points should be considered for testing by microsatellite instability (MSI) and/or immunohistochemical staining of four MMR proteins (MLH1, MSH2, MSH6, and PMS2). 

Two international consortia, C4CMMRD and international BMMRD consortium have proposed CMMRD surveillance guidelines (Table 1). 

**Table 1:** Surveillance protocol for CMMRD patients. CMMRD, constitutional mismatch repair deficiency

Examination	Start age	Frequency	Tumors	Comment
MRI brain	At diagnosis	Q 6 months	Brain tumors	Should not be replaced with WB MRI
WB MRI	6 years	Once a year	All tumors	Should not replace dedicated CNS imaging
CBC	1 year	Q 6 months	Leukemia	May be considered
Abdominal US	1 year	Q 6 months	Lymphoma	May be considered/ can be alternated with WB MRI
Upper GI endoscopy, VCE, ileocolonoscopy	4-6 years	Once a year	GI tumors	Upper and lower endoscopy, to be increased once polyps are found
GYN exam, urine cytology, dipstick	20 years	Once a year	Genitourinary tumors	As per LS guidelines

Surveillance for hematological, gastrointestinal, and brain tumors begins at early childhood while screening for genitourinary neoplasms is reserved for older children [8-9]. Full blood count is used for leukemia surveillance. Dedicated MRI brain is performed for CNS tumors. Whole body MRI (WB MRI) is recommended as in Li Fraumeni syndrome [10], for surveillance of other cancers that may arise in this cancer predisposing condition to better understand spectrum of CMMRD neoplasms. Rapid identification of neoplasms in patients and their siblings can help in genetic counseling, earlier diagnosis, and better outcomes with prompt treatment. 

Several therapeutic prospects arise while managing these patients that include potential toxicity of chemotherapeutic agents to the host, resistance to conventional agents [11-14], potential for targeted approaches [14-15], and role of tumor maturing agents in chemoprevention [8, 16-17]. These prospects are questions for future research and may provide better understanding of treatment modalities for these children and their families. 

## Conclusions

Constitutional mismatch repair deficiency is a rare autosomal recessive syndrome associated with wide spectrum of malignancies. The spectrum of cancers occurring in these patients and their ages of presentation is yet to be studied in detail. Our case adds to the literature that colorectal cancer can occur in these patients during early adolescent age. Sibling screening and subsequent genetic counseling is important in countries like us where consanguinity is much prevalent. 
